# The Missing Link in Head and Neck Oncology: Integrating Prosthodontic Rehabilitation From Day 1

**DOI:** 10.7759/cureus.88217

**Published:** 2025-07-18

**Authors:** Shubham K Srivastava, Shitij Srivastava, Abhinav Shekhar, Akshim Rana, Chinmoy Sikdar

**Affiliations:** 1 Department of Prosthodontics, Sardar Patel Post Graduate Institute of Dental and Medical Sciences, Lucknow, IND

**Keywords:** maxillofacial prosthetics, multidisciplinary cancer care, oral oncology, prosthodontic rehabilitation, quality of life

## Abstract

Head and neck cancers often require aggressive treatments such as surgery, radiotherapy, and chemotherapy, which, although effective in tumor control, frequently lead to debilitating anatomical and functional impairments. Speech disturbances, impaired mastication, and facial disfigurement severely affect patients' quality of life. Despite its critical role in restoring these vital functions, prosthodontic rehabilitation remains underutilized, especially in low- and middle-income countries. Maxillofacial prosthodontists provide essential services, including obturators, speech aids, and facial prostheses, yet they are rarely included in the early phases of oncology treatment planning. Delayed referral often results in limited prosthetic success due to tissue fibrosis and psychological burden. Early involvement, ideally at diagnosis or preoperative planning, enables better anatomical preservation, timely impressions, and improved outcomes through immediate rehabilitation strategies. Barriers to integration include a lack of awareness among oncologists, poor interdisciplinary communication, limited infrastructure, and insufficient training in oncologic prosthodontics. In regions like India, this is exacerbated by a shortage of trained specialists and uneven access to care. Educational reforms and policy initiatives are urgently needed to standardize early prosthodontic referral and expand rehabilitative services. This editorial underscores the pressing need for systemic change to incorporate prosthodontists as integral members of oncology teams. Ensuring timely, multidisciplinary care can restore not only physical function but also psychological well-being and social confidence, thus redefining survivorship through a more holistic approach.

## Editorial

Head and neck cancers are among the most devastating malignancies, often necessitating complex oncologic therapies including extensive surgical resection, radiation, and chemotherapy. These life-saving interventions, while effective in achieving tumor control, frequently leave patients with significant anatomical and functional deficits. Impairments in mastication, deglutition, speech, and facial esthetics are common and profoundly impact the patient's psychosocial well-being and quality of life. Yet despite these consequences, prosthodontic rehabilitation is frequently omitted from early treatment planning, relegated instead to a delayed, often neglected, post-treatment phase [1].

Prosthodontic rehabilitation, particularly maxillofacial prosthodontics, plays a vital role in restoring function and improving outcomes in patients with head and neck cancer. This specialty addresses the complex functional and esthetic challenges resulting from tumor resection and adjunctive therapies. Maxillofacial prosthodontists are uniquely trained to design and fabricate obturators for maxillary defects, mandibular guidance prostheses, palatal lift and speech aid prostheses, and custom facial prostheses for disfigured or missing external facial structures [2]. These devices significantly enhance patients' ability to eat, speak, and interact socially. However, such interventions are often delayed until well after surgical recovery or radiation therapy, when tissue changes such as fibrosis and scarring reduce prosthetic adaptability and patient compliance [3].

A proactive, multidisciplinary approach is essential. Integrating prosthodontic planning into the early stages of oncology care, ideally from the point of diagnosis, offers numerous advantages. Collaborative treatment planning involving surgeons, radiation oncologists, and prosthodontists ensures that resections are executed in ways that optimize prosthetic rehabilitation. For example, immediate surgical obturators can be fabricated and delivered at the time of maxillary resection, preserving function and reducing psychological trauma [4]. Presurgical impressions, implant placement planning, and clear communication about reconstructive priorities can dramatically improve both short- and long-term outcomes [3].

Despite these clear benefits, several barriers continue to hinder early prosthodontic involvement. There is a pervasive lack of awareness among surgical and medical oncologists regarding the scope and value of prosthodontic rehabilitation. Interdisciplinary communication is often insufficient, and many cancer treatment centers, especially in low- and middle-income countries, lack the infrastructure or trained personnel to provide comprehensive prosthodontic services. In India, for instance, the number of maxillofacial prosthodontists is disproportionately low compared to the prevalence of oral cancer [1]. Moreover, undergraduate and postgraduate dental education in many regions provides limited exposure to oncologic rehabilitation, resulting in an underprepared workforce to handle these complex cases [2].

Addressing these challenges requires systemic change. First, cancer treatment centers should institutionalize multidisciplinary tumor boards that include prosthodontists as core team members. Early referrals and collaborative planning must become standard practice rather than exceptions. Second, dental education curricula must be revised to incorporate oncology-focused prosthodontic training, both at the graduate and postgraduate levels. Continuing education programs and fellowships in maxillofacial prosthodontics should be expanded to increase the number of trained professionals [5]. Third, policy initiatives must recognize prosthodontic rehabilitation as an essential health service in cancer care, deserving of public funding and infrastructure investment [1].

Importantly, patient-centered care demands a focus beyond survival. Survivorship should be defined not only by the absence of disease but by the restoration of function, esthetics, dignity, and the ability to lead a meaningful life. Ignoring the rehabilitative phase of cancer care leads to incomplete treatment and avoidable suffering. Early prosthodontic intervention empowers patients, enhances their quality of life, and supports holistic recovery [4].

In conclusion, prosthodontic rehabilitation should no longer be viewed as a secondary or elective service in oncology. It must be integrated into the primary treatment pathway from day 1. Doing so will bridge a long-standing gap in cancer care, elevate the standard of interdisciplinary collaboration, and ensure that patients not only survive cancer but also regain the confidence to eat, speak, smile, and live with dignity.

## References

[1] Dugad JA, Athavale S, Chouksey G, Tongya R (2017). Oral cancer rehabilitation: requirement and responsibility of maxillofacial prosthodontists. Surg Rehabil.

[2] Vosselman N, Alberga J, Witjes MH, Raghoebar GM, Reintsema H, Vissink A, Korfage A (2021). Prosthodontic rehabilitation of head and neck cancer patients—challenges and new developments. Oral Dis.

[3] Salinas TJ (2010). Prosthetic rehabilitation of defects of the head and neck. Semin Plast Surg.

[4] Quadri MF, Alamir AW, John T, Nayeem M, Jessani A, Tadakamadla SK (2020). Effect of prosthetic rehabilitation on oral health-related quality of life of patients with head and neck cancer: a systematic review. Transl Cancer Res.

[5] Rathee M, Alam M, Malik S (2022). Role of maxillofacial prosthodontist as a member of interdisciplinary oncology team in oral and maxillofacial rehabilitation: a brief review. Int J Head Neck Surg.

